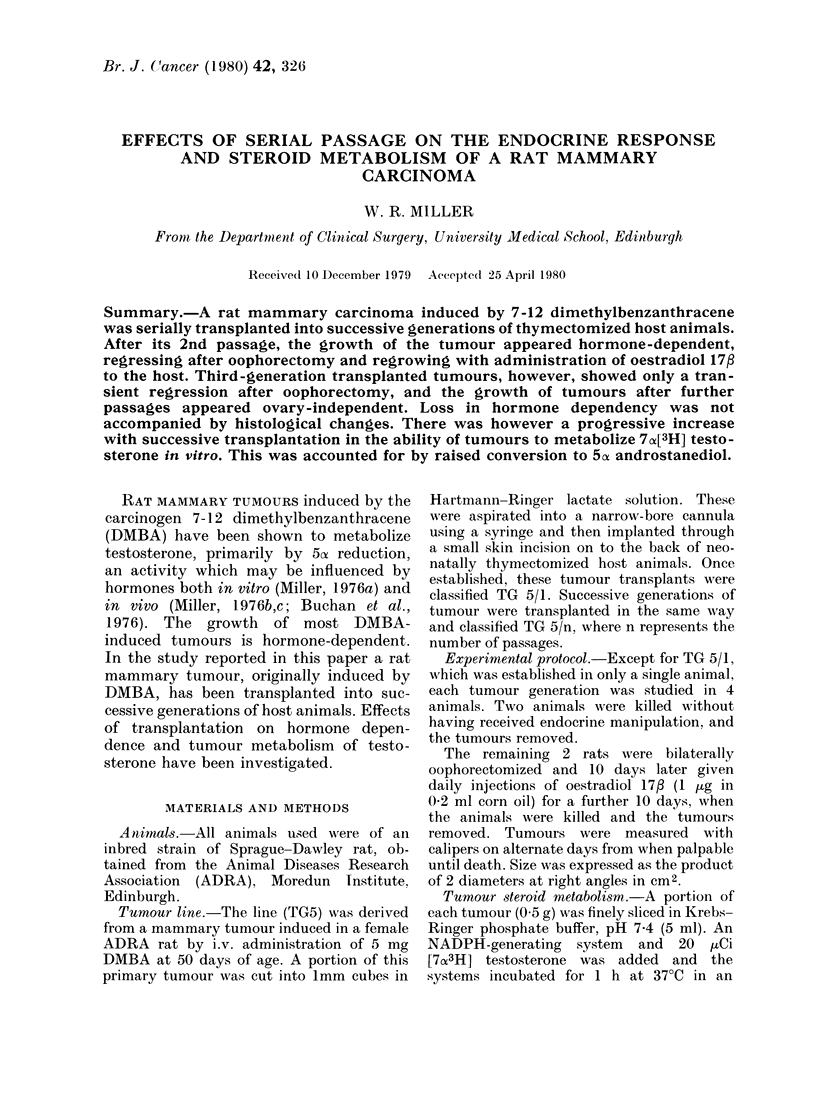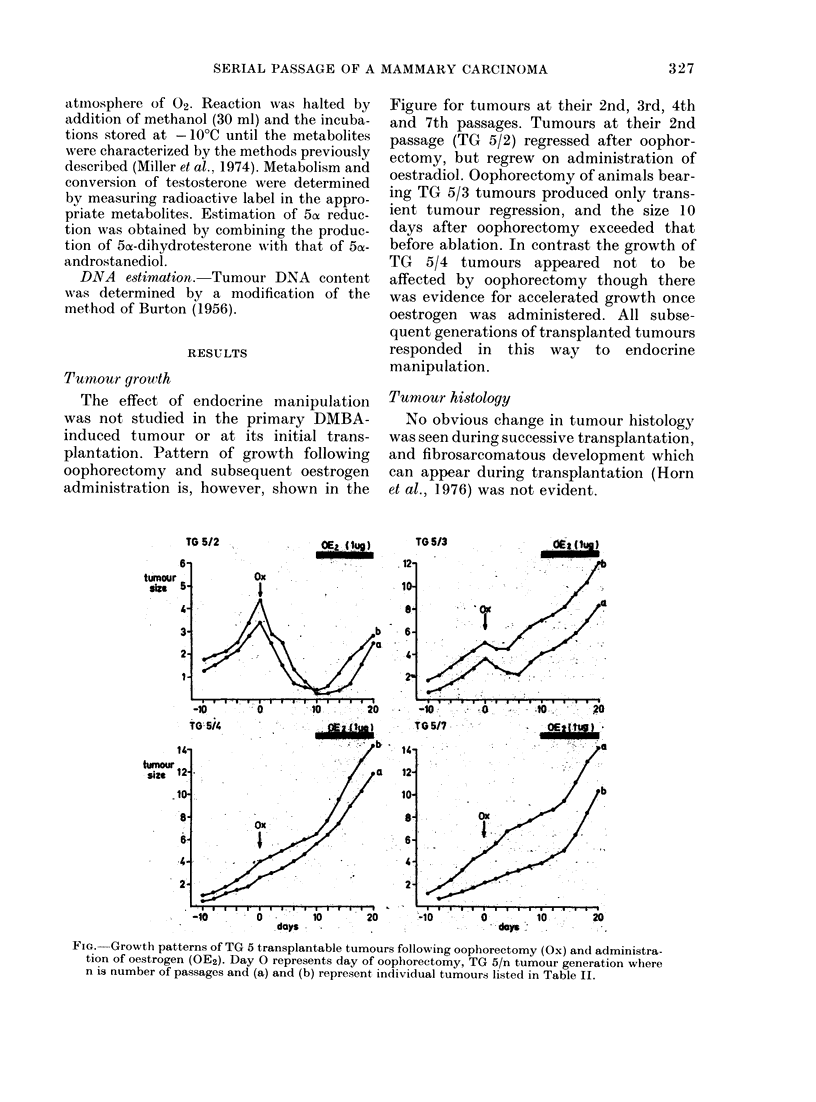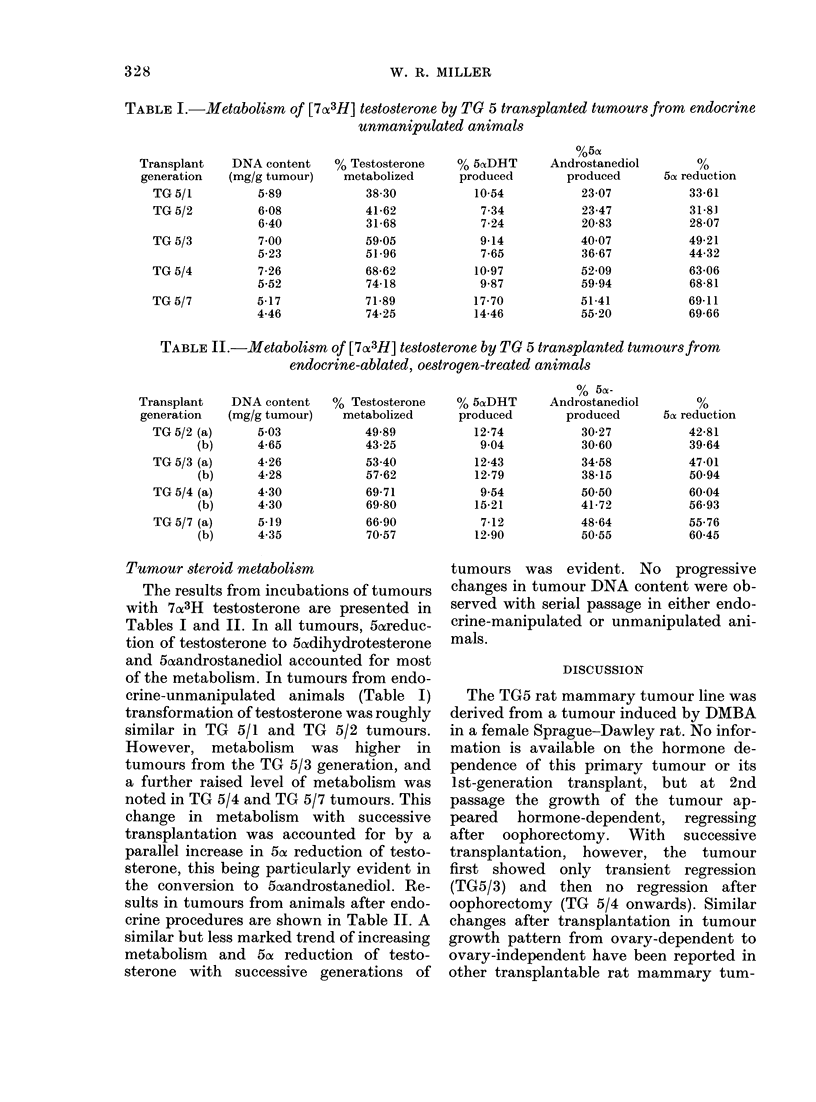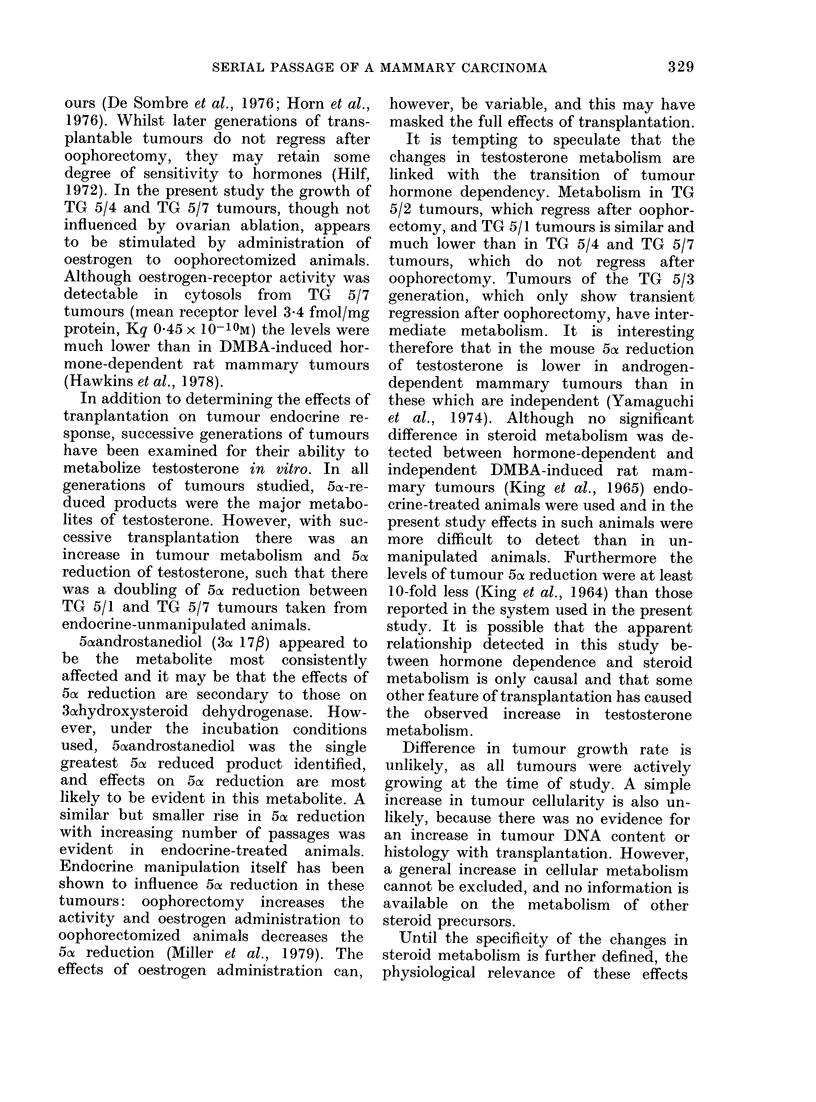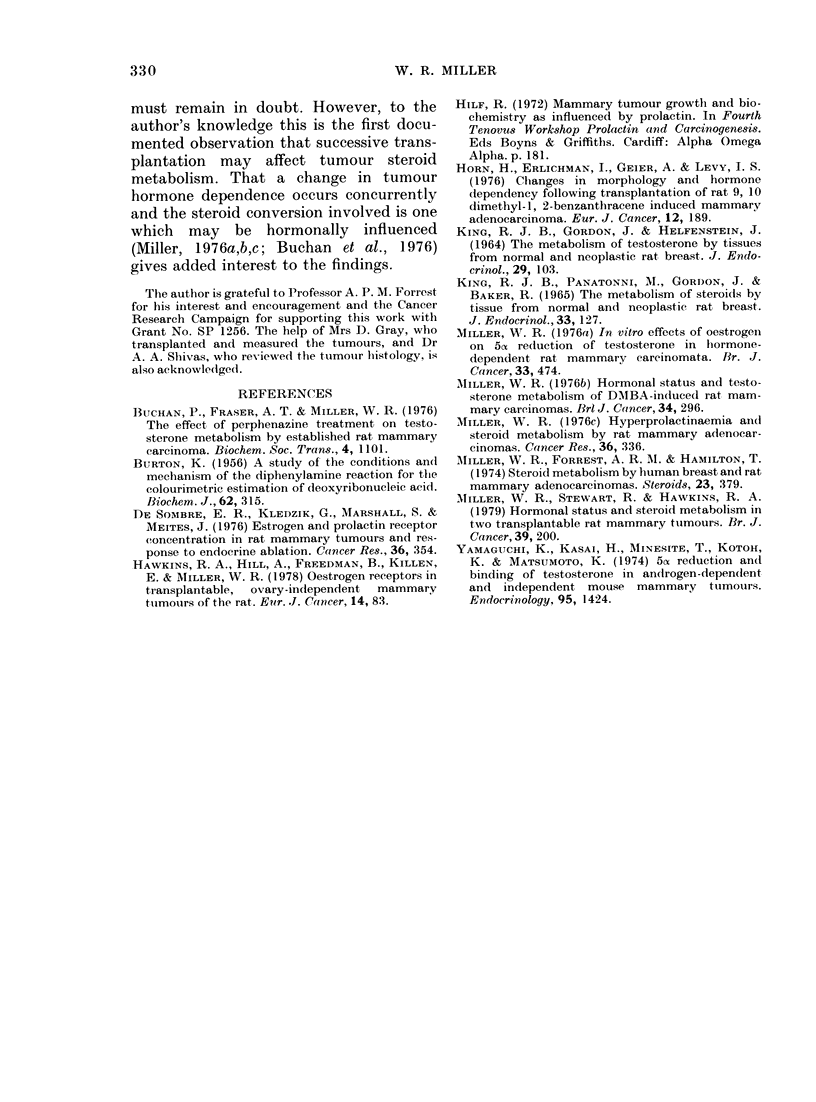# Effects of serial passage on the endocrine response and steroid metabolism of a rat mammary carcinoma.

**DOI:** 10.1038/bjc.1980.234

**Published:** 1980-08

**Authors:** W. R. Miller

## Abstract

A rat mammary carcinoma induced by 7-12 dimethylbenzanthracene was serially transplanted into successive generations of thymectomized host animals. After its 2nd passage, the growth of the tumour appeared hormone-dependent, regressing after oophorectomy and regrowing with administration of oestradiol 17 beta to the host. Third-generation transplanted tumours, however, showed only a transient regression after oophorectomy, and the growth of tumours after further passages appeared ovary-independent. Loss in hormone dependency was not accompanied by histological changes. There was however a progressive increase with successive transplantation in the ability of tumours to metabolize 7 alpha [3H] testosterone in vitro. This was accounted for by raised conversion to 5 alpha androstanediol.


Br. J. Cancer (1980) 42, 326

### EFFECTS OF SERIAL PASSAGE ON THE ENDOCRINE RESPONSE AND STEROID METABOLISM OF A RAT MAMMARY CARCINOMA

W. R. MILLER

*From the Department of Clinical Surgery, University Medical School, Edinburgh*

Received 10 December 1979 Accepted 25 April 1980

**Summary.—A rat mammary carcinoma induced by 7-12 dimethylbenzanthracene was serially transplanted into successive generations of thymectomized host animals. After its 2nd passage, the growth of the tumour appeared hormone-dependent, regressing after oophorectomy and regrowing with administration of oestradiol 17$\beta$ to the host. Third-generation transplanted tumours, however, showed only a transient regression after oophorectomy, and the growth of tumours after further passages appeared ovary-independent. Loss in hormone dependency was not accompanied by histological changes. There was however a progressive increase with successive transplantation in the ability of tumours to metabolize 7$\alpha$[$^3$H] testosterone *in vitro*. This was accounted for by raised conversion to 5$\alpha$ androstanediol.**

RAT MAMMARY TUMOURS induced by the carcinogen 7-12 dimethylbenzanthracene (DMBA) have been shown to metabolize testosterone, primarily by 5$\alpha$ reduction, an activity which may be influenced by hormones both *in vitro* (Miller, 1976*a*) and *in vivo* (Miller, 1976*b,c*; Buchan *et al.*, 1976). The growth of most DMBA-induced tumours is hormone-dependent. In the study reported in this paper a rat mammary tumour, originally induced by DMBA, has been transplanted into successive generations of host animals. Effects of transplantation on hormone dependence and tumour metabolism of testosterone have been investigated.

### MATERIALS AND METHODS

*Animals.*—All animals used were of an inbred strain of Sprague–Dawley rat, obtained from the Animal Diseases Research Association (ADRA), Moredun Institute, Edinburgh.

*Tumour line.*—The line (TG5) was derived from a mammary tumour induced in a female ADRA rat by i.v. administration of 5 mg DMBA at 50 days of age. A portion of this primary tumour was cut into 1mm cubes in Hartmann–Ringer lactate solution. These were aspirated into a narrow-bore cannula using a syringe and then implanted through a small skin incision on to the back of neonatally thymectomized host animals. Once established, these tumour transplants were classified TG 5/1. Successive generations of tumour were transplanted in the same way and classified TG 5/n, where n represents the number of passages.

*Experimental protocol.*—Except for TG 5/1, which was established in only a single animal, each tumour generation was studied in 4 animals. Two animals were killed without having received endocrine manipulation, and the tumours removed.

The remaining 2 rats were bilaterally oophorectomized and 10 days later given daily injections of oestradiol 17$\beta$ (1 $\mu$g in 0·2 ml corn oil) for a further 10 days, when the animals were killed and the tumours removed. Tumours were measured with calipers on alternate days from when palpable until death. Size was expressed as the product of 2 diameters at right angles in cm$^2$.

*Tumour steroid metabolism.*—A portion of each tumour (0·5 g) was finely sliced in Krebs–Ringer phosphate buffer, pH 7·4 (5 ml). An NADPH-generating system and 20 $\mu$Ci [7$\alpha^3$H] testosterone was added and the systems incubated for 1 h at 37°C in an atmosphere of $O_2$. Reaction was halted by addition of methanol (30 ml) and the incubations stored at $-10°C$ until the metabolites were characterized by the methods previously described (Miller *et al.*, 1974). Metabolism and conversion of testosterone were determined by measuring radioactive label in the appropriate metabolites. Estimation of $5\alpha$ reduction was obtained by combining the production of $5\alpha$-dihydrotestesterone with that of $5\alpha$-androstanediol.

*DNA estimation.*—Tumour DNA content was determined by a modification of the method of Burton (1956).

### RESULTS

### *Tumour growth*

The effect of endocrine manipulation was not studied in the primary DMBA-induced tumour or at its initial transplantation. Pattern of growth following oophorectomy and subsequent oestrogen administration is, however, shown in the Figure for tumours at their 2nd, 3rd, 4th and 7th passages. Tumours at their 2nd passage (TG 5/2) regressed after oophorectomy, but regrew on administration of oestradiol. Oophorectomy of animals bearing TG 5/3 tumours produced only transient tumour regression, and the size 10 days after oophorectomy exceeded that before ablation. In contrast the growth of TG 5/4 tumours appeared not to be affected by oophorectomy though there was evidence for accelerated growth once oestrogen was administered. All subsequent generations of transplanted tumours responded in this way to endocrine manipulation.

### *Tumour histology*

No obvious change in tumour histology was seen during successive transplantation, and fibrosarcomatous development which can appear during transplantation (Horn *et al.*, 1976) was not evident.

FIG.—Growth patterns of TG 5 transplantable tumours following oophorectomy (Ox) and administration of oestrogen ($OE_2$). Day O represents day of oophorectomy, TG 5/n tumour generation where n is number of passages and (a) and (b) represent individual tumours listed in Table II.

TABLE I.—*Metabolism of* [$7\alpha^3H$] *testosterone by TG 5 transplanted tumours from endocrine unmanipulated animals*

| Transplant generation | DNA content (mg/g tumour) | % Testosterone metabolized | % 5αDHT produced | %5α Androstanediol produced | % 5α reduction |
|---|---|---|---|---|---|
| TG 5/1 | 5·89 | 38·30 | 10·54 | 23·07 | 33·61 |
| TG 5/2 | 6·08 | 41·62 | 7·34 | 23·47 | 31·81 |
| | 6·40 | 31·68 | 7·24 | 20·83 | 28·07 |
| TG 5/3 | 7·00 | 59·05 | 9·14 | 40·07 | 49·21 |
| | 5·23 | 51·96 | 7·65 | 36·67 | 44·32 |
| TG 5/4 | 7·26 | 68·62 | 10·97 | 52·09 | 63·06 |
| | 5·52 | 74·18 | 9·87 | 59·94 | 68·81 |
| TG 5/7 | 5·17 | 71·89 | 17·70 | 51·41 | 69·11 |
| | 4·46 | 74·25 | 14·46 | 55·20 | 69·66 |

TABLE II.—*Metabolism of* [$7\alpha^3H$] *testosterone by TG 5 transplanted tumours from endocrine-ablated, oestrogen-treated animals*

| Transplant generation | DNA content (mg/g tumour) | % Testosterone metabolized | % 5αDHT produced | % 5α-Androstanediol produced | % 5α reduction |
|---|---|---|---|---|---|
| TG 5/2 (a) | 5·03 | 49·89 | 12·74 | 30·27 | 42·81 |
| (b) | 4·65 | 43·25 | 9·04 | 30·60 | 39·64 |
| TG 5/3 (a) | 4·26 | 53·40 | 12·43 | 34·58 | 47·01 |
| (b) | 4·28 | 57·62 | 12·79 | 38·15 | 50·94 |
| TG 5/4 (a) | 4·30 | 69·71 | 9·54 | 50·50 | 60·04 |
| (b) | 4·30 | 69·80 | 15·21 | 41·72 | 56·93 |
| TG 5/7 (a) | 5·19 | 66·90 | 7·12 | 48·64 | 55·76 |
| (b) | 4·35 | 70·57 | 12·90 | 50·55 | 60·45 |

*Tumour steroid metabolism*

The results from incubations of tumours with $7\alpha^3H$ testosterone are presented in Tables I and II. In all tumours, 5αreduction of testosterone to 5αdihydrotestosterone and 5αandrostanediol accounted for most of the metabolism. In tumours from endocrine-unmanipulated animals (Table I) transformation of testosterone was roughly similar in TG 5/1 and TG 5/2 tumours. However, metabolism was higher in tumours from the TG 5/3 generation, and a further raised level of metabolism was noted in TG 5/4 and TG 5/7 tumours. This change in metabolism with successive transplantation was accounted for by a parallel increase in 5α reduction of testosterone, this being particularly evident in the conversion to 5αandrostanediol. Results in tumours from animals after endocrine procedures are shown in Table II. A similar but less marked trend of increasing metabolism and 5α reduction of testosterone with successive generations of tumours was evident. No progressive changes in tumour DNA content were observed with serial passage in either endocrine-manipulated or unmanipulated animals.

### DISCUSSION

The TG5 rat mammary tumour line was derived from a tumour induced by DMBA in a female Sprague–Dawley rat. No information is available on the hormone dependence of this primary tumour or its 1st-generation transplant, but at 2nd passage the growth of the tumour appeared hormone-dependent, regressing after oophorectomy. With successive transplantation, however, the tumour first showed only transient regression (TG5/3) and then no regression after oophorectomy (TG 5/4 onwards). Similar changes after transplantation in tumour growth pattern from ovary-dependent to ovary-independent have been reported in other transplantable rat mammary tumours (De Sombre *et al.*, 1976; Horn *et al.*, 1976). Whilst later generations of transplantable tumours do not regress after oophorectomy, they may retain some degree of sensitivity to hormones (Hilf, 1972). In the present study the growth of TG 5/4 and TG 5/7 tumours, though not influenced by ovarian ablation, appears to be stimulated by administration of oestrogen to oophorectomized animals. Although oestrogen-receptor activity was detectable in cytosols from TG 5/7 tumours (mean receptor level 3·4 fmol/mg protein, $Kq$ $0{\cdot}45 \times 10^{-10}$M) the levels were much lower than in DMBA-induced hormone-dependent rat mammary tumours (Hawkins *et al.*, 1978).

In addition to determining the effects of tranplantation on tumour endocrine response, successive generations of tumours have been examined for their ability to metabolize testosterone *in vitro*. In all generations of tumours studied, 5α-reduced products were the major metabolites of testosterone. However, with successive transplantation there was an increase in tumour metabolism and 5α reduction of testosterone, such that there was a doubling of 5α reduction between TG 5/1 and TG 5/7 tumours taken from endocrine-unmanipulated animals.

5αandrostanediol (3α 17β) appeared to be the metabolite most consistently affected and it may be that the effects of 5α reduction are secondary to those on 3αhydroxysteroid dehydrogenase. However, under the incubation conditions used, 5αandrostanediol was the single greatest 5α reduced product identified, and effects on 5α reduction are most likely to be evident in this metabolite. A similar but smaller rise in 5α reduction with increasing number of passages was evident in endocrine-treated animals. Endocrine manipulation itself has been shown to influence 5α reduction in these tumours: oophorectomy increases the activity and oestrogen administration to oophorectomized animals decreases the 5α reduction (Miller *et al.*, 1979). The effects of oestrogen administration can, however, be variable, and this may have masked the full effects of transplantation.

It is tempting to speculate that the changes in testosterone metabolism are linked with the transition of tumour hormone dependency. Metabolism in TG 5/2 tumours, which regress after oophorectomy, and TG 5/1 tumours is similar and much lower than in TG 5/4 and TG 5/7 tumours, which do not regress after oophorectomy. Tumours of the TG 5/3 generation, which only show transient regression after oophorectomy, have intermediate metabolism. It is interesting therefore that in the mouse 5α reduction of testosterone is lower in androgen-dependent mammary tumours than in these which are independent (Yamaguchi *et al.*, 1974). Although no significant difference in steroid metabolism was detected between hormone-dependent and independent DMBA-induced rat mammary tumours (King *et al.*, 1965) endocrine-treated animals were used and in the present study effects in such animals were more difficult to detect than in unmanipulated animals. Furthermore the levels of tumour 5α reduction were at least 10-fold less (King *et al.*, 1964) than those reported in the system used in the present study. It is possible that the apparent relationship detected in this study between hormone dependence and steroid metabolism is only causal and that some other feature of transplantation has caused the observed increase in testosterone metabolism.

Difference in tumour growth rate is unlikely, as all tumours were actively growing at the time of study. A simple increase in tumour cellularity is also unlikely, because there was no evidence for an increase in tumour DNA content or histology with transplantation. However, a general increase in cellular metabolism cannot be excluded, and no information is available on the metabolism of other steroid precursors.

Until the specificity of the changes in steroid metabolism is further defined, the physiological relevance of these effects must remain in doubt. However, to the author's knowledge this is the first documented observation that successive transplantation may affect tumour steroid metabolism. That a change in tumour hormone dependence occurs concurrently and the steroid conversion involved is one which may be hormonally influenced (Miller, 1976*a*,*b*,*c*; Buchan *et al.*, 1976) gives added interest to the findings.

The author is grateful to Professor A. P. M. Forrest for his interest and encouragement and the Cancer Research Campaign for supporting this work with Grant No. SP 1256. The help of Mrs D. Gray, who transplanted and measured the tumours, and Dr A. A. Shivas, who reviewed the tumour histology, is also acknowledged.

### REFERENCES

BUCHAN, P., FRASER, A. T. & MILLER, W. R. (1976) The effect of perphenazine treatment on testosterone metabolism by established rat mammary carcinoma. *Biochem. Soc. Trans.*, **4**, 1101.

BURTON, K. (1956) A study of the conditions and mechanism of the diphenylamine reaction for the colourimetric estimation of deoxyribonucleic acid. *Biochem. J.*, **62**, 315.

DE SOMBRE, E. R., KLEDZIK, G., MARSHALL, S. & MEITES, J. (1976) Estrogen and prolactin receptor concentration in rat mammary tumours and response to endocrine ablation. *Cancer Res.*, **36**, 354.

HAWKINS, R. A., HILL, A., FREEDMAN, B., KILLEN, E. & MILLER, W. R. (1978) Oestrogen receptors in transplantable, ovary-independent mammary tumours of the rat. *Eur. J. Cancer*, **14**, 83.

HILF, R. (1972) Mammary tumour growth and biochemistry as influenced by prolactin. In *Fourth Tenovus Workshop Prolactin and Carcinogenesis.* Eds Boyns & Griffiths. Cardiff: Alpha Omega Alpha. p. 181.

HORN, H., ERLICHMAN, I., GEIER, A. & LEVY, I. S. (1976) Changes in morphology and hormone dependency following transplantation of rat 9, 10 dimethyl-1, 2-benzanthracene induced mammary adenocarcinoma. *Eur. J. Cancer*, **12**, 189.

KING, R. J. B., GORDON, J. & HELFENSTEIN, J. (1964) The metabolism of testosterone by tissues from normal and neoplastic rat breast. *J. Endocrinol.*, **29**, 103.

KING, R. J. B., PANATONNI, M., GORDON, J. & BAKER, R. (1965) The metabolism of steroids by tissue from normal and neoplastic rat breast. *J. Endocrinol.*, **33**, 127.

MILLER, W. R. (1976*a*) *In vitro* effects of oestrogen on 5α reduction of testosterone in hormone-dependent rat mammary carcinomata. *Br. J. Cancer*, **33**, 474.

MILLER, W. R. (1976*b*) Hormonal status and testosterone metabolism of DMBA-induced rat mammary carcinomas. *Brl J. Cancer*, **34**, 296.

MILLER, W. R. (1976*c*) Hyperprolactinaemia and steroid metabolism by rat mammary adenocarcinomas. *Cancer Res.*, **36**, 336.

MILLER, W. R., FORREST, A. R. M. & HAMILTON, T. (1974) Steroid metabolism by human breast and rat mammary adenocarcinomas. *Steroids*, **23**, 379.

MILLER, W. R., STEWART, R. & HAWKINS, R. A. (1979) Hormonal status and steroid metabolism in two transplantable rat mammary tumours. *Br. J. Cancer*, **39**, 200.

YAMAGUCHI, K., KASAI, H., MINESITE, T., KOTOH, K. & MATSUMOTO, K. (1974) 5α reduction and binding of testosterone in androgen-dependent and independent mouse mammary tumours. *Endocrinology*, **95**, 1424.